# RhoGTPases as Key Players in Mammalian Cell Adaptation to Microgravity

**DOI:** 10.1155/2015/747693

**Published:** 2015-01-29

**Authors:** Fiona Louis, Christophe Deroanne, Betty Nusgens, Laurence Vico, Alain Guignandon

**Affiliations:** ^1^INSERM U1059, Laboratoire de Biologie du Tissu Osseux, Université Jean Monnet, 42023 Saint-Etienne Cedex, France; ^2^Laboratoire de Biologie des Tissus Conjonctifs, GIGA, Université de Liège, 4000 Sart Tilman, Belgium

## Abstract

A growing number of studies are revealing that cells reorganize their cytoskeleton when exposed to conditions of microgravity. Most, if not all, of the structural changes observed on flown cells can be explained by modulation of RhoGTPases, which are mechanosensitive switches responsible for cytoskeletal dynamics control. This review identifies general principles defining cell sensitivity to gravitational stresses. We discuss what is known about changes in cell shape, nucleus, and focal adhesions and try to establish the relationship with specific RhoGTPase activities. We conclude by considering the potential relevance of live imaging of RhoGTPase activity or cytoskeletal structures in order to enhance our understanding of cell adaptation to microgravity-related conditions.

## 1. Introduction

Microgravity has been demonstrated to have profound effects on both cellular and molecular levels, including changes in cell morphology [1, 2], alterations of proliferation, growth or differentiation [3, 4], modification of gene expression [5–7], and changes in signal transduction cascades [5, 8]. Single undifferentiated cells* in vitro* respond to altered conditions of gravity, but not all sensors and upstream regulators are known, which limits our understanding of cell sensitivity to microgravity-related conditions and even more to microgravity per se.

There are numerous observations strengthening the idea that cytoskeletal structures and cell surface receptors connected to them play an important role in the regulation of the differentiation potential of stem cells [9]. As changes of shape and of the inner cytoskeletal architecture are common cell responses under conditions of real or simulated microgravity [2], the idea of cytoskeletal involvement in the cellular response to microgravity seems obvious. Moreover, stem cells or multipotent cells are recognized as being sensitive to mechanical stresses, which are known to influence cell commitment [10, 11]. The idea that not only terminally differentiated cells but also multipotent cells are sensitive to microgravity explains why even limited effects on cell commitment could have dramatic consequences. Small GTPases of the Rho family are known to control several aspects of cell dynamics (vesicular transport, traffic, cytoskeleton turnover) [12, 13] and appear to be the key players when trying to gain a better understanding of the effects of microgravity on differentiated and multipotent cells.

This review first attempts to highlight the fact that structures involved in mechanotransduction pathways are responsible for adaptation to microgravity: it will be explained that structural changes observed in cells exposed to real and simulated microgravity may result from specific RhoGTPase regulations. Then, the degree to which the effects of microgravity are important controllers of multipotent cell commitment will be discussed, highlighting the critical role of RhoGTPases in these regulations. The monitoring of RhoGTPase activities in conditions of microgravity is still a challenge as it is a dynamic process that controls other highly dynamic processes such as actin polymerization or focal adhesion turnover. In order to decipher cell adaptation in conditions of microgravity, the community is in need of a live imaging technology, like the one from Pache et al. [15], but that can be set up in flight! We are conscious of all the difficulties of using Förster resonance energy transfer- (FRET-) based biosensors dedicated to RhoA (Ras homolog gene family member A) and Rac1 (Ras-related C3 botulinum toxin substrate 1), two important actors of this GTPases family, under conditions of microgravity, and we are convinced that research groups that are successful with these types of sensors will provide very exciting results that will eliminate many confounding factors related to conditions of microgravity, such as launch vibrations. We predict that many specific GAP and GEF (resp., RhoGTPases inhibitors and stimulators) will turn out to be key players in cell adaptation to microgravity-related conditions in the future.

## 2. Mechanotransductors as Gravity Sensors

Discussions of whether an* in vitro* single cell or a cell population can sense changes in the gravitational field are very controversial. The currently most unknown research area involves the mechanism by which the physical event of g-force susception (by invagination, sedimentation, or buoyancy) becomes the biological process of g-force perception. Despite this, an enormous body of experimental data undoubtedly indicates that several types of cultured cells are sensitive to gravity [16, 17]. If, in fact, cells do not fall (collapse), it is because they are supported in some way. This support takes the form of a mechanical stress, set up by the intermolecular forces in response to the distortion produced by gravity. In conditions where gravity is limited (microgravity) (such as those found in an orbiting vehicle) there is thus no distortion produced, and consequently, there is no (limited) mechanical stress.

It seems that undifferentiated cells have structural elements that may play the role of “gravitational sensors” and “sense” the intensity of a mechanical tension and that several intracellular processes can depend on the value of the gravitational force. Theoretical considerations suggest that the forces involved are too small to trigger any response to the changed environment. Several research teams think that these effects are mostly caused by changes at the tissue and organ level [17] and that such environmental changes are stronger and more diverse [18] (e.g., lung, heart, and kidney become larger while spleen or pancreas get smaller in rats [19]). In conclusion, gravitational effects have been considered significant for cells with a diameter of no less than 10 *μ*m [20]. Thus, microgravity seems to alter mammalian cells as compared to bacterial cells which are normally too small.

Actors in the mechanotransduction chain represent key elements involved in microgravity adaptation. Nature provides clear examples of defined mechanoreceptors in eukaryotes such as the statoliths in plants and the otoliths of the inner ear in most species of vertebrates. Similar specialized cells of the sense organs detect pressure (touch) and vibrations and communicate these physical stimulations to the nerves of the afferent pathway up to the brain.

It thus seems that undifferentiated mammalian cells do indeed have structural elements that may play the role of a “gravitational sensor” and “sense” the intensity of a mechanical tension and that many intracellular processes (adhesion, proliferation, survival, contractility, migration, extracellular matrix (ECM) architecture, gene expression, etc.) can depend on the intensity of the gravitational force. The identification of cell structures capable of acting as gravisensors in* in vitro* cells still remains a problem. The general view of mechanosensing is that the overall cell is sensitive and is not a particular element.

In our opinion, the most significant element (*primum movens*) that may impact on cytoskeletal dynamics under microgravity is the displacement of the nucleus. The location of the nucleus is probably dictated by a tension equilibrium between the cyto- and nucleoskeletons and we can imagine that these tensions are constantly changing (in response to signals) and that the nucleus probably oscillates continuously [21]. A microgravity environment may influence the oscillating behavior of the nucleus [22] and then trigger a series of mechanical adjustments that may modulate cell shape and structures, as well as functions by way of transcription activities.

In response to changes in nucleus location, cytoskeletal structures and integrins might be solicited for cell adaptation. The cytoskeleton is a network of three interconnected systems of filaments: the actin microfilaments, the microtubules, and the intermediate filaments. They condition the shape of the cells and the major mechanical functions such as adhesion, polarization, directional migration, as well as proliferation, survival, or apoptosis, gene expression, and architectural organization of their supporting scaffold [12].

Experiments in real and simulated conditions of microgravity have shown that cytoskeletal modulations can occur quickly after variations in gravity have taken place. Numerous articles have reported on changes within 30 min of the onset of a microgravity simulation, affecting from focal adhesions to signal transduction. Nevertheless cell response can be observed only after few seconds following gravitational changes, for example, in parabolic flight experiments. After only 22 seconds of microgravity, ML-1 thyroid cancer cells showed no sign of apoptosis or necrosis, but the F-actin and cytokeratin cytoskeleton was altered [23]. Endothelial cells also demonstrated no signs of death (after 31 parabolas of 22 seconds) but had a cytoplasmic rearrangement and an alteration of cytoskeleton gene expressions [24]. Concerning mesenchymal stem cells, morphologic characteristics of apoptosis cells (cell shrinkage, membrane blebbing, nuclear chromatin condensation, etc.) and decreased cell viability (rate of apoptosis up to 56.95%) were reported 12 h after parabolic flight experiment. The F-actin stress fibers and microtubules were disrupted and the expression of p53 (mRNA and protein levels) was upregulated [25]. So, gravity-induced response of cells can occur very early, within seconds.

The reorganization of the cytoskeleton is believed to govern the modifications in size and shape of cells and nuclei as well as the patterning, number, and maturation of focal adhesions. The structures of the cytoskeleton, nuclei, and integrins may claim, to varying degrees, to fulfill the role of gravisensors [26].

The most likely candidates to assume the role of these structures are various elements of the cytoskeleton, the nucleus, intracellular organelles, and also certain cell surface receptors (integrins), which interact both with cytoskeletal structures and the extracellular matrix. These structures are able to sense constraints and deformations in the matrix which are caused either by a gravitational or mechanical field and convert this signal into intracellular messengers, which then give rise to a cellular response to the changes in gravity [21, 27]. It is also noteworthy that the cytoskeleton and integrins are not the primary sensors but react in response to their regulatory proteins (controllers of polymerization/destabilization agent).

Numerous cellular processes are controlled by gravity, for example, calcium signaling, mechanotransduction, ligand-receptors interactions, and cell-cell communications, which are all linked [28]. During these mechanisms, cell density is important because force transmission is greatest at cell-cell and cell-substrate focal contacts where signaling molecules are concentrated or clustered (i.e., integrin clustering) [17]. Indeed, transmission of forces from outside the cell through cell-matrix and cell-cell contacts appears to control the maturation or disassembly of these adhesions which rearrange the organization and contractile activity of the cytoskeleton. The cytoskeletal tensions formed at adhesions mediate mechanical signalling [29]. Thus, vinculin phosphorylation determines whether cadherins transmit force and can produce biologically distinct functions [30].

In microgravity, gravity-induced breakage of cell-cell adhesions is reduced. So, cell-cell interaction was shown to be promoted in absence of gravity [31]. Cell adhesion protein expression, specifically proteins found in tight junctions and adherens junctions, was upregulated resulting in enhanced cell-cell contact between cells (endothelial cells [32]). Also, increased levels of E-cadherin were observed in 3D tumor constructs cultured in simulated microgravity [33].

In osteoblasts, a downregulation of cell-cell adhesion proteins, such as catenin, is observed [34] and also a reduction in adhesion proteins such as vinculin and extracellular matrix proteins such as fibronectin [35]. To explain this phenomenon, Levenberg et al. showed that there is an autoregulatory pathway that is activated by the presence of cell-cell or cell-substrate adhesion sites. So, when cell-cell adhesion is enhanced, cell-matrix adhesion is decreased [36]. These adhesion processes are also dependent on Ca^2+^ signaling pathways, such as cell-cell adhesion via E-cadherin. This Ca^2+^ dependence is through activation of the protein kinase C (PKC) second messenger system, as well as activation of phospholipase C (PLC), which in turn activates a signaling cascade, resulting in the release of intracellular Ca^2+^ [37]. This release of intracellular calcium, facilitating the binding of cadherins and *β*-catenin to the actin filaments comprising the cytoskeleton, resulted in increased strength of cell-cell contacts [38].

And several teams actually found a calcium release in vascular smooth muscle cells after 14 days of hindlimb unloading [39] and a downregulation of Calcium channel after 28 days [40]. Also, a reduction in intracellular calcium concentration is observed after 2 days of simulated microgravity in chondrocytes [41] as well as in neurons [42]. Moreover, in neutrophils, PKC pathway is inhibited under microgravity leading to a decrease in intracellular concentration of Ca^2+^ [43].

**Figure 1:**
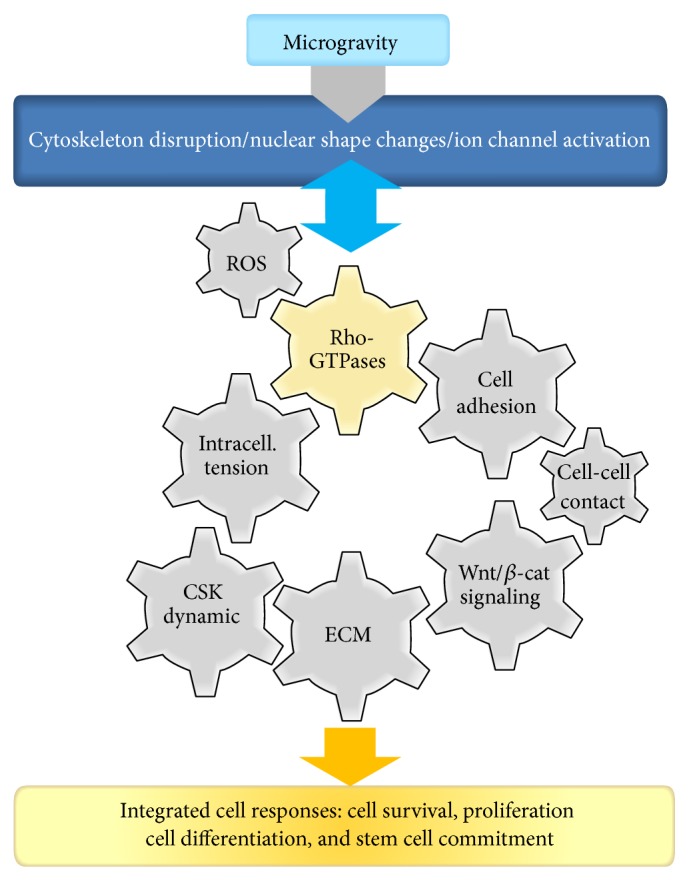
Central role of the RhoGTPases in the integrated response of mammalian cell to microgravity-related conditions. A growing number of studies are revealing that cells reorganize their cytoskeleton, modulate intracellular tension, and initiate nuclear shapes changes when exposed to conditions of microgravity. Most, if not all, of the structural changes observed on flown cells can be explained by modulation of RhoGTPases, which are mechanosensitive switches. RhoGTPases are known for cytoskeletal dynamics control; nevertheless they are also involved in many other aspects as discussed in this review. We identify general principles dependent on RhoGTPases and define cell sensitivity to gravitational stresses such as oxidative stress, intracellular tension, cell-cell and cell-ECM adhesions, and Wnt/*β*-catenin pathways. We will try to establish that integrated cellular responses in microgravity are related to specific RhoGTPase activities.

All the structural changes observed in cells subjected to microgravity-related conditions are dictated/controlled by dynamic molecular switches of the GTPase family (Figure 1). Small RhoGTPases mainly control the regulation of intracellular traffic and are responsible for cytoskeletal dynamics [44].

## 3. RhoGTPases: Mechanosensitive Molecular Switches

**Figure 2:**
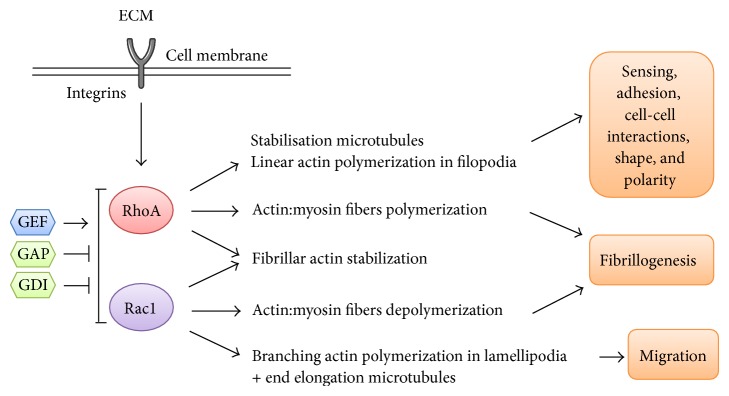
RhoGTPase actions on the cytoskeleton and cell dynamics (modified from [14]). Integrins are necessary for translating the mechanical properties of the extracellular environment into intracellular RhoGTPase-signaling pathways. RhoA influences filopodia formation and focal adhesion assembly and maturation, in addition to controlling stress fiber formation and intracellular tension. Rac1 primarily controls actin assembly and formation of lamellipodia to ensure cell migration. Fibrillogenesis is controlled positively by RhoA and negatively by Rac1. Both RhoA and Rac1 are controlled by specific activators (GEF) and inhibitors (GAP, GDI). Cell adaptation to mechanical/gravitational challenges triggers activation of pathways integrated by RhoGTPases.

RhoGTPases, found in all eukaryotic cells, are key regulatory molecules which link surface receptors to the organization and turnover of the cytoskeleton, govern the formation of cell-matrix adhesions, and uphold the transcriptional control of gene expression, cell survival, and proliferation [45]. They are members of the Ras superfamily of small GTP-binding proteins and are divided into three major classes: RhoA, Rac1, and Cdc42. GTPases are molecular switches that use a simple biochemical strategy to control complex cellular processes. They switch between two conformational states: a guanosine triphosphate- (GTP-) bound (“active”) state and another (“inactive”) state related to guanosine diphosphate (GDP). In their inactive forms, RhoGTPases are sequestrated in the cytoplasm, while upon signaling identified by integrins and growth factor receptors, they switch to their active forms and translocate to the cell membrane [46]. There, they activate distinct and specific effector molecules which in turn regulate the organization of the cytoskeleton and cell-matrix adhesions, thus controlling cellular activities such as adhesion, and also affect cell proliferation and the expression of specific genes (Figure 2) [12]. The cycle between the active and inactive forms is under the direct control of three groups of regulatory proteins. The guanine nucleotide exchange factors (GEFs) catalyze the exchange of GDP for GTP to activate Rho proteins. The Rho proteins are then deactivated by GTPase-activating proteins (GAPs) which increase the intrinsic GTPase activity of the Rho protein, leading to the hydrolysis of GTP to GDP. The third group of proteins involved in the cycle of Rho signaling is guanine dissociation inhibitors (RhoGDI), which hide the isoprenyl groups of GTPases, an action that promotes the sequestration of inactive GTPases in the cytosol. The RhoGDIs also inhibit the release of GDP from the GTPase and contribute to the maintenance of GTPases in an inactive state. The Rho protein cycle is stimulated by agonists acting through G protein-coupled receptors (GPCRs), tyrosine kinase receptors, cytokine receptor activation, and mechanical stresses that mainly govern the activity of the GEFs [47]. The best known actions of the RhoGTPases on mechanical parameters of the cytoskeleton can be underscored by the expression of constitutively active RhoA and Rac1 in cell lines. These modelsshow that RhoA activation leads to better cell spreading but lower mechanical properties, while Rac1 activation induces mechanotransduction [48]. As we assume that exposure to gravitational stress is a mechanical stimulation, Rac1 might be rapidly induced in microgravity-related conditions. These results reveal the importance of RhoGTPases on mechanosensing, cell shape adaptation, or signal transduction. We will summarize below the different controls they can have on cellular mechanisms and metabolism.

## 4. RhoGTPases Control Cytoskeleton Dynamic

In microgravity, a qualitative and quantitative analysis of the structures of F-actin, *β*-tubulin, and vinculin has revealed a higher density of filamentous actin and a decreased organization in stress fibers. Exposing mesenchymal stem cells (MSCs) to low gravity affected the distribution of the different filaments and more specifically led to a significant reduction of the F-actin fibers [49, 50], extended filopodia, increased perinuclear distribution, and decreased density [15, 51]. Moreover, other research groups have found evidence of an accumulation of actin at the cell border [52, 53]. This loss of stress fibers is accompanied by an increase in monomeric G-actin content within the cells. The preceded alterations may be explained by a preferential reduction of RhoA activity.

Indeed, the activation of RhoA or Rac1 leads to the assembly of contractile actin:myosin filaments, protrusive actin-rich lamellipodia, and protrusive actin-rich filopodia, which in turn give rise to both the formation (actin polymerization) and the organization (filament bundling) of actin filaments. Thus, a number of studies (e.g., [54]) have shown that Rho kinase (ROCK) modulates the nonmuscle myosin II (NMM-II) activity by phosphorylation. Another protein, cofilin, regulates actin polymerization and filament elongation. Its phosphorylation leads to inactivation and occurs primarily through LIM kinases (LIMK), which are activated by Rac1-dependent kinases. Moreover, LIMK-dependent phosphorylation of cofilin can also be induced by RhoA acting through its target ROCK, which may be an important event in the stabilization of actin:myosin filaments [55]. Microgravity leads to an alteration of the actin cytoskeleton and consequently to a decrease of integrin signaling that may be caused by the inhibition of RhoA activity. The absence of gravity increases the G-actin form, which reduces cofilin phosphorylation, and is consistent with a decrease in focal adhesions and thus stress fibers [56].

Finally, if a constitutively active RhoA is overexpressed, a recovery stress of the fibers is enabled, similar to what can be observed under normal gravity, and integrin signaling is restored as shown in MSCs [57].

Microtubules play critical roles in eukaryotic cells. They are key structural elements of the mitotic spindle apparatus during mitosis and interphase and serve as tracks upon which motor proteins transport vesicles and other components move throughout the cell [58]. Several studies have mentioned perinuclear clustering in the microtubular network during microgravity [50, 59]. Also, the loss of the radial structure of microtubules has been observed after long stretches of time (4 h) in microgravity [60].

Microgravity has also been proposed to influence microtubules by affecting the self-organization of filaments. According to the theory on self-organization and in a series of* in vitro* studies with a change in gravity direction [61, 62] and microgravity [61], it was clearly shown that microtubule self-organization is sensitive to the direction and the magnitude of gravity, which may explain the results obtained under microgravity. Furthermore, the observed disorganization of microtubules may lead to a reduced rate of chromosome segregation during mitosis, while alterations of actin microfilaments and focal adhesions may also slow down cytokinesis and thus cell proliferation.

RhoGTPases regulate microtubule dynamics in different ways. Rac1 can phosphorylate at Ser16 of the microtubule plus-end-binding proteins (stathmins), which occurs in response to a number of extracellular stimuli [63]. The effect of RhoA on microtubule dynamics is likely to be context-dependent. For instance, in migrating fibroblasts, RhoA promotes the formation of stabilized microtubules. Also, microtubules play a major role in defining cell shape and polarity through the specific interaction of their plus-ends with proteins at the cell cortex. This plus-end capture of microtubules has been attributed to a number of plus-end-binding proteins, whose activities are influenced by RhoGTPases [12]. Altogether, results on microtubules observed in conditions of microgravity may be explained by an alteration of the RhoA and Rac1 activities.

Microgravity has also had an impact on intermediate filaments, which after 12 min in microgravity appeared as large bundles and aggregates in the vimentin network, that is, the most distributed of all intermediate filament proteins [64]. ROCK phosphorylates intermediate filament proteins, specifically at the cleavage furrow during cytokinesis. This cleavage furrow-specific phosphorylation plays an important role in the breakdown of local intermediate filaments and enables an efficient separation of intermediate filament networks [65]. In fact, RhoA and Rac1 induce phosphorylation and reorganization of vimentin through kinases such as RhoA-associated protein kinase 2 (ROCK2), p21-activated kinase (PAK), Src kinase (family of nonreceptor tyrosine kinases), and tyrosine kinases [66].

Concerning lamins, which are nuclear intermediate filaments, Uva et al. showed DNA fragmentation in glial cells after 30 min of microgravity and explained the phenomenon by caspases causing lamina to collapse and chromatin to condense [67]. Proteins linking nucleoskeleton and cytoskeleton complexes (LINC), thus connecting lamina to the cytoskeleton, have been found. When it comes to laminopathy models, in which this LINC complex is disrupted, they lead mostly to RhoA inhibition and lowered cytoplasmic elasticity, while actin and focal adhesion structures are mildly affected [68]. Changes in nuclear structures, that we identified earlier as an important initiator of microgravity effects [22], might explain the RhoA activity inhibition and changes in cell tension evoked under microgravity.

Rac1 was shown to accumulate in the nuclear envelope in addition to being expressed in the nucleoplasm and seemed to have the same pattern as that reported for lamin B [69]. This Rac1 accumulation was proven to promote cell division. In microgravity, the altered proliferation observed by Dai et al. or Damm et al. [70, 71] is controversial since Yuge et al. [72] rather found an increased proliferation in human mesenchymal stem cells. We thus suggest, based on our results obtained on rat osteosarcoma [73], that the lower proliferation might be explained by a reduced Rac1 activity in conditions of microgravity.

## 5. RhoGTPases as Regulators of Cell Adhesion and Matrix Remodeling

Integrins are transmembrane receptors that mediate the attachment between a cell and its surroundings, such as other cells or the ECM. In signal transduction, integrins convey information about the chemical composition and mechanical status of the environment into the cell. Therefore, in addition to transmitting mechanical forces, they are involved in cell signaling and the regulation of cell cycles, shapes, and motility [74].

Among the ligands of integrins can be mentioned fibronectin, vitronectin, collagen, and laminin. Then, adapter proteins such as talin and vinculin link the cytoskeleton to integrins, which attach the cell to the substrate, forming a focal adhesion. A variety of signaling proteins are associated with focal adhesions, including focal adhesion kinase (FAK), which is an important mediator of signaling at these centers. Forces are also transmitted to the substrate at these sites. In fibroblasts, local forces correlate with the area of focal adhesions and actomyosin contractility blocking results in a rapid disruption of focal adhesions [75].

In conditions of microgravity, a reduced focal adhesion-related area (frequently reported [35, 76]) can be explained by the lower tension applied to the cytoskeleton. This situation can be associated with an inactivation of RhoA, and as a result by decreased fibrillogenesis (fibronectin collagen) dramatically limiting integrin signaling. The proof of a reduced integrin signaling is that MSCs have been observed to display changes in the expression levels of collagen-specific integrins after 7 days of cultivation in a rotational bioreactor [77]. In fact, activated expression of the*α*2-integrin has been seen during the course of MSC differentiation to osteogenesis [53]. In addition, Loesberg et al. found a downregulation of*α*1, *β*1, and *β*3 integrins after 48 h of simulated microgravity [78].

*β*1 integrin has been shown to be important for mediating the response of MSCs to mechanical stimulation [79]. Upon application of fluid shear stress, an increase in alkaline phosphatase (ALP) activity and expression of osteogenic markers is observed, along with the activation of FAK and extracellular signal-regulated kinase 1/2 (ERK1/2). But when *β*1 integrins are blocked, FAK and ERK1/2 activation becomes inhibited [79]. Phosphorylation of FAK has also been demonstrated to be important for osteogenic differentiation of human MSCs in response to tension [80]. In microgravity-related conditions, the limitation of integrin signaling can be a plausible explanation for the reduced osteogenesis.

In addition, limitation of the integrin-mediated response can also reduce important negative regulatory pathways. Thus, growth of preadipocytes on a fibronectin matrix inhibits adipocyte differentiation and this effect is overcome when actin filaments are disrupted and promotes a rounding-up of cells [81]. However, *β*1 in association with*α*5 binds to fibronectin, and Liu et al. [82] reported the presence of an expression switch from*α*5 to*α*6 at the growth arrest stage of differentiation, which is consistent with an ECM change observed during adipogenesis. This switch is necessary in order to go from proliferation to differentiation of preadipocytes and can be explained by integrins*α*6*β*1 that bind to laminin and can thus interfere with chromatin and gene regulation.

These two integrins*α*5 and*α*6 are coordinately regulated by cyclic adenosine monophosphate (cAMP). Interestingly, cAMP has been shown to be activated in microgravity [83–85]. RhoA and cAMP have antagonistic roles in regulating cellular morphology [86]. Thus, the excessive production of cAMP in microgravity may explain the limitation of RhoA activation during adipogenesis followed by the integrin switch of*α*5 to*α*6 to promote adipogenesis. Also, it is well established that cAMP enhances the expression of both CCAAT-enhancer-binding proteins (C/EBP)*α* and *β* [87, 88] and initiates adipogenesis via the transcription factor CREB (cAMP response element binding protein) [89].

Concerning Rac1, cell adhesion to fibronectin (*α*5 integrin) but not to laminin (*α*6 integrin) is particularly efficient in activating Rac1 [90], leading to osteogenesis via *β*-catenin/Wnt pathways [91]. In microgravity, fibrillogenesis is rapidly limited [92, 93], which explains the delay or absence of osteogenesis in multipotent cells. The extracellular domains of cadherins and *β*-catenin provide a link to*α*-catenin and the actin cytoskeleton [94]. Upon tyrosine phosphorylation, *β*-catenin also plays a significant role in signaling when translocated to the nucleus to regulate cell proliferation [95].

Noritake et al. [96] have explained the increase in Rac1 during osteogenesis: until subconfluence, cell adhesions accumulate E-cadherins at the sites of cell-cell contacts which induce Rac1, and thus actin-meshwork formation and *β*-catenin, leading to osteogenesis. In fact, before E-cadherin-mediated cell-cell adhesion is established, GDP-Rac1 is sequestered in the cytosol by Rho GDI. When E-cadherins accumulate, GDP-Rac1 is converted to GTP-Rac1, through the action of a GEF, and is targeted to the plasma membrane releasing *β*-catenin linked to E-cadherin, which can go to the nucleus [97].

In addition, cell-to-cell physical contact via N-cadherin also plays a crucial role in regulating osteoblastic activity such as alkaline phosphatase activity and *β*-catenin signaling [98, 99]. Consequently, reduced cell-cell adhesion observed in microgravity, due to limited proliferation, may induce a decrease in Rac1 action and osteogenesis.

Moreover, it has been largely described that matrix rigidity affects osteogenesis. MSCs grown on collagen-I stiff gels (linking to*α*1 or*α*2-*β*1 integrins) have demonstrated activated osteogenesis, whereas softer collagen-I gels prime MSCs for a myogenic lineage [100]. However, cytoskeleton and the dynamic mechanical balance that exists between cells and their ECM support appear as major players in several mechanotransduction pathways [74]. Microgravity decreases the expression of collagen I [101–103], induces matrix metalloproteinases (MMP) production, and reduces the level of fibrillar collagen. Thus, it could be expected that altered conditions of gravity may change the mechanical properties of ECM (i.e., the stiffness). Several studies, for example, McBeath et al. or Shih et al. [104, 105], have shown that osteogenic differentiation becomes increased on stiffer matrices, as evident by type-I collagen, osteocalcin, Runx2 gene expressions, ROCK, FAK, and ERK1/2 induction and alizarin red S staining for mineralization. Consequently, FAK affects osteogenic differentiation through ERK1/2, whereas RhoA and ROCK regulate both FAK and ERK1/2 [105].

In microgravity, an initial modification of cytoskeletal dynamics might be at the origin of the following vicious circle: remodeling of a cytoskeleton is associated with a reduced internal tension (contractility) leading to the dispersion of FA. With such a reduction in FA, the cell tension cannot be restored and fibrillogenesis might be limited. Matrix deposition limitation and MMP activation (Rac1 dependent process [106, 107]) may further reduce the matrix stiffness, thus amplifying the dispersion of FA and reducing cell tension and fibrillogenesis. After a short exposure (from minutes to hours) to microgravity-related conditions (before fibrillogenesis, MMP production), the matrix stiffness is not modified. We can thus speculate that the ability of the cells to detect the stiff matrix they are normally seeded on has become rapidly impaired. Mechanical information is normally conveyed by ECM and cells by FA adaptation following tensegrity principles (equilibrium of internal and external tension) [21]; in microgravity it seems that the displacement of the nucleus (sensitive to G) conveys the mechanical stimulus and from a tensegrity perspective, the cell adapts to the reduced tension by lowering the ECM tension (interruption of fibrillogenesis and MMP production). The short-term adaptation of the cell to microgravity that we have described up to now seems to be characterized by a rapid reduction of RhoA and an increased Rac1 activity. Altogether, these studies revealed that the control of cytoskeleton remodeling by RhoGTPases impacts on cell adhesion signaling, limiting internal cellular tension as well as ECM fibrillogenesis, and triggers MMP production, thus limiting cell-matrix adhesion and survival.

## 6. RhoGTPases in Stem Cell Commitment

In simulated microgravity, cellular morphology is drastically changed after 7 days. The MSCs appear rounder and less firmly attached to their substrate than under conditions of normal gravity. Rather, they are very spread out and display a fibroblastic morphology [53].

Since the work by McBeath et al., we know that the shape of a cell affects its differentiation potential [104]. Thus, MSCs that have been allowed to adhere over a larger area are able to differentiate towards the osteogenic lineage while cells adhering to a smaller area are restricted to the adipogenic lineage. These impacts on lineage commitment by mesenchymal stem cells seem to be regulated by shape-induced changes in the RhoGTPase activity and cytoskeletal tension [108]. Yao et al. [109] showed that the cell shape itself is an inherent cue to regulate stem cell differentiation, both with and without external chemical induction factors. Thus, according to McBeath et al. [104], expressing dominant-negative RhoA causes MSCs to become adipocytes, while constitutively active RhoA induces osteoblastic or myocytic differentiation [110, 111].

Concerning Rac1, it has been shown to promote cell adhesion and spreading and thereby to prevent the cell shape change and the establishment of the cortical actin structure necessary for adipocyte formation [109]. Adhering cells are characterized by an elaborate network of stress fibers and focal adhesions and are thus more prone to adopt a fibroblastic cell shape reflecting cytoskeleton tension [112, 113], which seems to be altered in conditions of microgravity.

The cell shape may also depend on the available substrate area and hence the cell density. However, if cellular growth is reduced in microgravity, the cell density will also be altered. Gao et al. [110] found that levels of RhoA activity did not vary substantially, but that the Rac1 activity was significantly higher in well-spread cells during early differentiation than in high-density cells.

They also demonstrated that Rac1 is necessary for osteogenesis and that constitutively active Rac1 inhibited adipogenesis, even if it is important for adipose commitment. Liu et al. [82] showed that an increase in preadipocyte density inhibited the RhoA activity and that a downregulation of the RhoA-ROCK pathway was required for both adipose lineage commitment and maturation [104, 111]. An increased cell density thus appeared to be critically important.

GTPases have also been shown to act in the cell cycle, mitosis, and cytokinesis. RhoGTPases influence the cyclin-dependent kinase (cdk) activity during the G1-Phase of the cell cycle. Thus, RhoGTPases control the organization of the microtubule and actin fibers during cell cycling. An inhibition of RhoA or Rac1 blocks the G1 progression in a variety of mammalian cell types [114, 115]. Also, Rac1 (but not RhoA) stimulates cyclin D1 transcription mediated by NF-*κ*B (nuclear factor kappa-light-chain-enhancer of activated B cells) [116, 117]. Thus, the necessity to downmodulate the Rac1 activity in adipogenesis is that Rac1 may prolong proliferation of preadipocytes, which is consistent with the reported effects of Rac1 on cyclin D1 [90, 118, 119]. In fact, Rac1 accumulates in the nucleus during the G2 phase of the cell cycle and promotes cell division [69]. Concerning the cell division itself, it has been shown that actin:myosin filaments, under the control of ROCK, are required at the cortex to allow positioning of the centrosomes [120]. RhoA also plays a crucial role in the contractile ring function [121].

**Figure 3:**
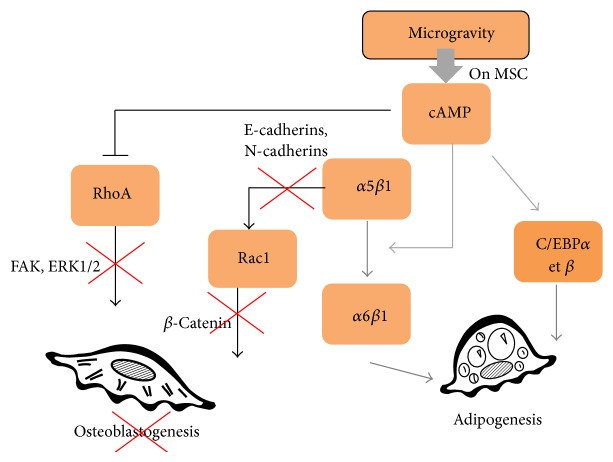
Role of AMPc on RhoGTPases activities and commitment of multipotent cells. Microgravity affects the growth, proliferation, and differentiation of multipotent cells by increasing AMPc production. AMPc contributes to cytoskeleton reorganization as it regulates negatively RhoA activity. Limitation of osteoblastogenesis might be linked to the ability of microgravity to reduce RhoA and Rac1 activities. RhoA and Rac1 activations support osteoblasts differentiation for their respective role in ERK activation and beta-catenin nuclear translocation. Sustained adipogenesis observed in microgravity-related condition might be linked to ability of AMPc to trigger integrin a5b1/a6b1 switch. Signaling through a6b1 integrin is known to support adipogenesis. A direct activation of adipogenic transcription factors (cEBPs) by AMPc has been also described.

Microgravity affects the growth, proliferation, and differentiation of osteoblasts. Since the inhibition of RhoA, observed under microgravity, blocks G1 progression [114, 115], this may explain the altered proliferation and differentiation of osteoblastic cells and increased adipogenesis as summarized in Figure 3.

Furthermore, several cytoskeletal components, including Rac1 GTPase activating protein 1 (Rac-GAP1) and Tropomodulin 1, segregate asymmetrically during stem cell division, and overexpression of these proteins may enhance MSC commitment, as already proven with asymmetrical divisions of hematopoietic stem cells to progenitor cells [122].

## 7. RhoGTPases and Wnt/*β*-Catenin Signaling Crosstalk

Three Wnt signaling pathways have been characterized: the canonical Wnt pathway, the noncanonical planar cell polarity pathway, and the noncanonical Wnt/calcium pathway. The canonical Wnt pathway leads to regulation of gene transcription, the noncanonical planar cell polarity pathway regulates the cytoskeleton via a RhoGTPase regulation that is responsible for the shape of the cell, and the noncanonical Wnt/calcium pathway regulates calcium inside the cell [123].

Mellor et al. found that Wnt signaling was inhibited in conditions of microgravity [124] and mouse osteoblasts subjected to simulated microgravity were found to have lower levels of several components of the Wnt/*β*-catenin signaling pathway. This may indicate, even indirectly, the activation of an adipogenic program under microgravity [125]. Moreover, Wan et al. [126] recently demonstrated a changed RhoA and *β*-catenin signaling after 1 and 2.5 h, respectively, in clinorotated osteoblasts. They revealed that both the RhoA activity and the TCF/LEF (T-cell factor-1 and lymphoid enhancing factor-1) activity, a *β*-catenin recruiter, were downregulated by unloading. However, the inhibition of *β*-catenin signaling blocked the unloading-induced RhoA suppression, and dominant negative RhoA inhibited the TCF/LEF suppression, revealing a regulation loop between *β*-catenin, RhoA, and TCF/LEF. Furthermore, while *β*-catenin signaling seemed to be required for microgravity regulation of RhoA, this response was not mediated by the actin cytoskeleton or intracellular tension [126]. The same was observed for Rac1/*β*-catenin signaling [91].

The Wnt canonical pathway involves the translocation of *β*-catenin to the nucleus, and *β*-catenin has been shown to promote osteogenic differentiation in early osteoblast progenitors* in vivo* [127]. In contrast, other studies have suggested that canonical Wnt signaling may actually promote stem cell renewal and inhibit osteogenic differentiation of osteoprogenitor cells* in vivo* [128], as well as promoting stem cell renewal in human MSCs derived from bone marrow [129]. Arnsdorf and colleagues [130] investigated the role of noncanonical Wnt signaling in mechanically induced osteogenic differentiation of MSCs. Exposure of MSCs to oscillatory fluid flow resulted in a translocation of *β*-catenin [131] and an upregulation of Wnt5a, which is capable of inducing both canonical and noncanonical pathways. Wnt5a is also necessary for the flow-induced activation of RhoA. However, the inhibition of Wnt5a did not affect the *β*-catenin translocation, which may instead be regulated by cadherin-catenin signaling. In addition, Santos et al. [132] showed that the activation of the RhoA/ROCK pathway by Wnt5a induced a downregulation of adipogenic markers. It was further reported that RhoA could also be activated by Wnt3a, one of the canonical Wnt family members [133], and that an inhibition of intracellular *β*-catenin decreased the RhoA activity [134].

Kim et al. [135] also found that Wnt signaling regulated the MSC differentiation into cardiomyocyte-like cells with a concomitant downregulation of RhoA and upregulation of Rac1. Concerning Rac1, it was shown to be a critical regulator in shear stress-driven *β*-catenin signaling in osteoblasts [91], and constitutively active Rac1 mutant caused a significant enhancement of the TCF/LEF activity.

These studies demonstrate that Wnt signaling is important for mechanically induced differentiation, through RhoA or Rac1 pathways. So, in conditions of microgravity, reduced RhoA, cell shape, and migratory behaviors can be explained by Wnt and *β*-catenin signaling. Finally, RhoGTPases are regulated by Wnt signaling, but in return, *β*-catenin location (translocation) is dependent on RhoGTPases. This intricate interplay between both regulatory elements makes them particularly important for the interpretation of microgravity effects.

## 8. RhoGTPases and Oxidative Stress

One of the first targets of Rac1 to be identified was p67phox, an essential structural component of the NADPH oxidase complex [136]. Since then, Rac1 has been reported to promote reactive oxygen species (ROS) production in many cells and to mediate the activity of the Nox family [137, 138]. Consequently, Rac1 activation leads to the generation of ROS enabling adipogenesis commitment [139] and reducing osteoblastogenesis [140, 141]. Moreover, GTPases act on the antioxidant master gene Nrf2 (nuclear factor-like 2), which activates a protective adaptive response to oxidative stress through transcriptional activation of antioxidant defense genes [142].

RhoA is involved in Nrf2 phosphorylation, which is necessary for its activation [143]. Nrf2 is a transcription factor for Hace1 (HECT domain and ankyrin repeat containing E3 ubiquitin protein ligase 1), and Hace1 binds and ubiquitylates Rac1 when the latter is associated with NADPH oxidase, thus blocking ROS generation by NOX [143, 144]. So, RhoA activation may limit ROS production and adipogenesis while Rac1 activation may support it. However, several research groups have reported that ROS causes RhoA activation [145, 146], while Nimnual et al. demonstrated that Rac1-mediated ROS production results in the downregulation of the RhoA activity [147]. This is also required for Rac1-induced formation of membrane ruffles and integrin-mediated cell spreading. The GTPase regulation by oxidative cell status thus still remains unclear.

In line with these papers, several research groups, such as Versari et al., have found increased oxidative stress during space flight due to microgravity [148, 149] and cosmic radiations [150]. As RhoA is decreased in microgravity, this could explain the increased production of reactive oxygen species. According to this paper, we can assume that Rac1 activities are increased in microgravity. An upregulated Rac1 activity fits well with enhanced ROS production and improved adipogenesis.

However, a higher Rac1 activity is also consistent with a higher ability for cell migration [151, 152]. Nevertheless, results of migration in space are controversial. Bone marrow cells from rats and human embryonic brain cells show a facilitated cell migration [153, 154], while bone marrow CD34+ cells have a lower migration potential in simulated microgravity [155]. We can interpret the apparent discrepancies in migration results based on the time spent in microgravity: for short-term exposure (from minutes to hours), there are several reasons to believe that RhoA is decreased and Rac1 increased in line with their reciprocal inhibition [156], but for longer exposure (from hours to days), the Rac1-induced ROS production may increase RhoA activation [145, 146] and reduce the Rac1 activity limiting migration capabilities. The missing information in microgravity is related to the lack of measurements of specific RhoGTPase activities.

## 9. RhoGTPases Activities Monitoring in Microgravity

**Figure 4:**
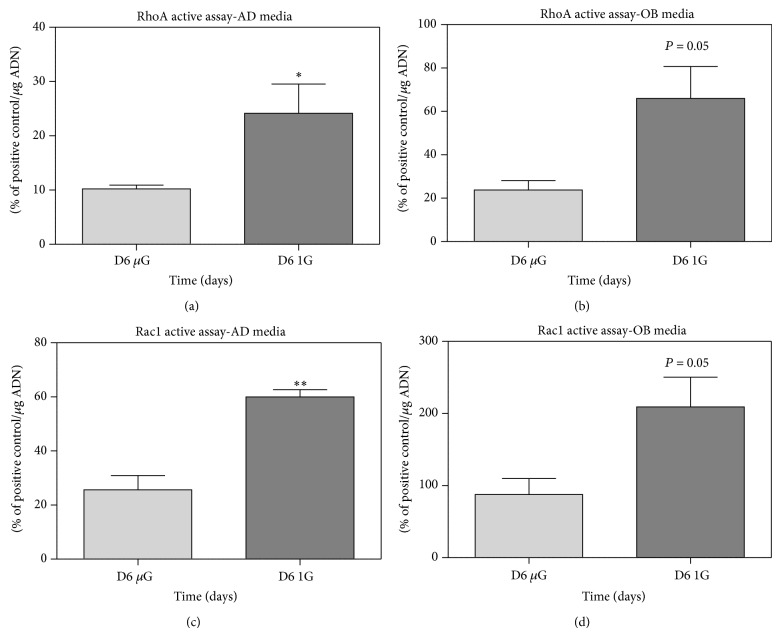
RhoA and Rac1 activities are downregulated after 6 days of culture in simulated-microgravity conditions. Cultures were performed with C3H10T1/2 (multipotent embryonic cells) on collagen-coated microbeads (Cytodex 3, Sigma) for adipogenic induction and on Cytodex 3 beads coated with apatite minerals complexed to collagen for an osteogenic one. The adipogenic media contained 1 *μ*M of rosiglitazone and the osteogenic media 5 mg/mL of L-ascorbic acid, *β*-glycerophosphate at 10^−3^ M, and retinoic acid at 10^−5^ M, in*α*MEM. Microbeads with cells were cultured for 2 days in 90 mm petri dishes (untreated for culture) with 10 mL of proliferation media (*α*MEM), after which the cells were switched 2 days in differentiated media, and finally left for 6 days in a NASA rotating wall vessel (RWV). In parallel, controls were realized by culturing beads in petri dishes. RhoA and Rac1 active assays were performed with specific G-LISA kits (cytoskeleton). The positive controls were pure active proteins of RhoA and Rac1 provided with the kit. The results are expressed as percentage of the positive controls; they show standard error of the mean (SEM) of samples extracted from three independent experiments and are compared with Student's statistical *t*-test.

Meyers et al. showed a reduction in active RhoA (−88% (±2%)) and a decrease in phosphorylation of cofilin after 7 days in microgravity, in addition to the absence of stress fibers [56]. If overexpression of active RhoA is carried out, this enables a recovery of stress fibers and restored integrin signals, similar to those observed in normal gravity in MSC [57]. In simulated microgravity, a decrease in RhoA activity was also observed after 72 h [157, 158]. Unfortunately nothing is known about Rac1 activity. Zayzafoon et al. thus proposed a model in which the cytoskeleton is actually not the first sensor, but a secondary step affected by a gravity-sensitive sensor. In this model, it is the RhoA inactivation that is followed by cytoskeletal changes and transduction at FAs [57], which explains the alterations on MSC differentiations observed in microgravity. To our knowledge, our team is the first to perform RhoA and Rac1 monitoring during osteogenesis and adipogenesis in simulated microgravity using embryonic mesenchymal stem cells. C3H10T1/2 multipotent cells were cultured in modeled microgravity using NASA's rotating wall vessels (RWV) or in control cultures under conditions of earth gravity for up to 8 days, seeded on collagen-coated microbeads (Cytodex 3, Sigma). The results presented in Figure 4 show significant decreases in both RhoA and Rac1 after long-term exposure to simulated microgravity. To our knowledge no comparison can be made with data obtained in real microgravity, unfortunately. Regardless of the limitation of the model when it comes to simulated microgravity-related conditions, these results clearly showed that downregulations of RhoA and Rac1 were compatible with enhanced adipogenesis and limited osteogenesis.

As preservation of active RhoGTPases in flight condition might be challenging, the recent validation of biosensors for imaging of active RhoA, Rac1, and Cdc42 represents an important step in understanding cell responses to microgravity. Despite the critical role of RhoGTPases that we describe in this review, there is a dramatic lack of data concerning the monitoring of their activities during exposure to microgravity particularly in real microgravity. These data are of crucial importance since cell adaptation is a dynamic process; we need to use available technologies such as fused fluorescent proteins and biosensors dedicated to following RhoGTPase activities in order to decipher cell adaptation in conditions of microgravity. On ground experiments, extensive biochemical and profiling studies on mechanotransduction pathways can be performed. In an automated spaceflight, the use of biosensors specific to molecules integrating many pathways such as RhoGTPases should be presented as a simplified and integrated view of cell mechanics. The community is in need of a live imaging data (already validated on ground [159]) that can be now used in flight conditions. We believe that groups that are successful in providing this type of integrated data will surprise our community whose thinking is limited by analysis of fixed images of cells and the monitoring of individual parameters.

## 10. Conclusion

**Figure 5:**
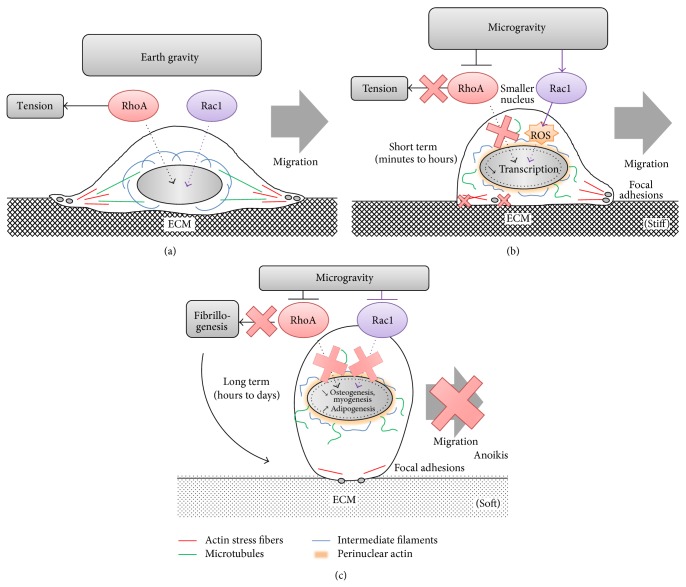
Proposed models describing the regulations of RhoA and Rac1 activities in space-related conditions. On Earth MSCs are well spread and exhibit a tensed cytoskeleton in particular of microtubules, intermediate filaments, and actin stress fibers associated with stable focal adhesions within the extracellular matrix. These elements are controlled by GTPases RhoA and Rac1. We hypothesize that during short-term exposure to microgravity, RhoA might be inhibited to allow cytoskeleton reorganization in respect to the new mechanical status. Cell tension reduction might be mandatory during this adaptation. At the same time, Rac1 is activated to control peripheral actin polymerization and induces ROS production. All these events lead rapidly to a rounder cell shape with disorganization of microtubules, stress fibers, intermediate filaments, and focal adhesions. Transcription may be also altered as nucleus shape is changed. In these conditions, cell is still able to migrate. When exposure to microgravity is prolonged both RhoA and Rac1 might be inhibited explaining decreases in osteogenesis and myogenesis and enhancement of adipogenesis of MSCs. In addition, RhoA inhibition limits fibrillogenesis (a tension-dependent process); extracellular matrix is not properly synthesized and lost its mechanical properties appearing softer for MSCs, reinforcing adipogenesis. At that time, migration is inhibited, consistent with cytoskeleton alterations and Rac1 decrease. MSCs become very round, have low adhesion, and may initiate anoikis.

RhoGTPases represent a unique hub for integration of biochemical and mechanical signals. As such, they are probably very rapidly involved in a cell's adaptation to microgravity-related conditions. Published data describing this adaptation have reported on alterations of the cytoskeleton, adhesion, and fibrillogenesis as well as an enhancement of the ROS production and migration that can be explained by the specific regulation of RhoGTPases. To summarize the literature, we can speculate that after a short exposure of a cell to microgravity, the RhoA activity is depressed and the Rac1 activity increased. For long-term exposure, osteogenesis has been reported to be impaired and adipogenesis promoted. These changes in multipotent cell commitment fit nicely with prolonged depressed activities of both RhoA and Rac1 (Figure 5).

As we are convinced that focal adhesion and F-actin fibers are not the primary sensors of microgravity-related signals (but rather transducers or effectors of the response), many specific GAP and GEF (resp., RhoGTPase inhibitors and stimulators) will emerge as new players in the adaptation of cells to microgravity-related conditions. What are the mechanisms that explain the activation or inhibition of these GTPases regulators? As we try to establish that mechanosensors are involved in cell adaptation to microgravity we can predict that critical players identified in these extreme conditions will in return be recognized in the mechanobiology field.

## Abbreviations

ALP: Alkaline phosphatase
C/EBP: CCAAT-enhancer-binding proteins
cAMP: Cyclic adenosine monophosphate
CREB: cAMP response element-binding protein
CSK: Cytoskeleton
ECM: Extracellular matrix
ERK1/2: Extracellular signal-regulated kinase 1/2
FAK: Focal adhesion kinase
FRET: Förster resonance energy transfer
GAPs: GTPase-activating proteins
GDIs: Guanine dissociation inhibitors
GDP: Guanosine diphosphate
GEFs: Guanine nucleotide exchange factors
GPCR: G protein-coupled receptor
GTP: Guanosine triphosphate
Hace1: HECT domain and ankyrin repeat containing E3 ubiquitin protein ligase 1
LIMK: LIM kinases
LINC: Proteins linking nucleoskeleton and cytoskeleton complexes
MMPs: Matrix metalloproteinases
MSC: Mesenchymal stem cell
NF-*κ*B: Nuclear factor kappa-light-chain-enhancer of activated B cells
NMM-II: Nonmuscle myosin II
Nrf2: Nuclear factor (erythroid-derived 2-) like 2
PAK: p21-activated kinase
Rac1: Ras-related C3 botulinum toxin substrate 1
RhoA: Ras homolog gene family, member A
ROCK: Rho kinase
ROCK2: RhoA-associated protein kinase 2
ROS: Reactive oxygen species
RWV: Rotating wall vessels
SEM: Standard error of the mean
Src family kinase: Family of nonreceptor tyrosine kinases
TCF/LEF: T-cell factor-1 (Tcf-1) and lymphoid enhancing factor-1 (Lef-1).
